# Climate Changes Exacerbate the Spread of *Ixodes ricinus* and the Occurrence of Lyme Borreliosis and Tick-Borne Encephalitis in Europe—How Climate Models Are Used as a Risk Assessment Approach for Tick-Borne Diseases

**DOI:** 10.3390/ijerph19116516

**Published:** 2022-05-27

**Authors:** Chrysa Voyiatzaki, Sevastiani I. Papailia, Maria S. Venetikou, John Pouris, Maria E. Tsoumani, Effie G. Papageorgiou

**Affiliations:** 1 Laboratory of Molecular Microbiology & Immunology, Department of Biomedical Sciences, University of West Attica, 12243 Athens, Greece; ml16068@uniwa.gr (S.I.P.); jopouris@uniwa.gr (J.P.); mtsoumani@uniwa.gr (M.E.T.); 2 Laboratory of Anatomy-Pathological Anatomy & Physiology Nutrition, Department of Biomedical Sciences, School of Health and Care Sciences, University of West Attica, 12243 Athens, Greece; mvenet@uniwa.gr; 3 Laboratory of Reliability and Quality Control in Laboratory Hematology (HemQcR), Department of Biomedical Sciences, School of Health and Care Sciences, University of West Attica, 12243 Athens, Greece; efipapag@uniwa.gr

**Keywords:** climate change, Europe, geographical distribution, *Ixodes ricinus*, tick-borne diseases, temperature

## Abstract

Climate change has influenced the transmission of a wide range of vector-borne diseases in Europe, which is a pressing public health challenge for the coming decades. Numerous theories have been developed in order to explain how tick-borne diseases are associated with climate change. These theories include higher proliferation rates, extended transmission season, changes in ecological balances, and climate-related migration of vectors, reservoir hosts, or human populations. Changes of the epidemiological pattern have potentially catastrophic consequences, resulting in increasing prevalence of tick-borne diseases. Thus, investigation of the relationship between climate change and tick-borne diseases is critical. In this regard, climate models that predict the ticks’ geographical distribution changes can be used as a predicting tool. The aim of this review is to provide the current evidence regarding the contribution of the climatic changes to Lyme borreliosis (LB) disease and tick-borne encephalitis (TBE) and to present how computational models will advance our understanding of the relationship between climate change and tick-borne diseases in Europe.

## 1. Introduction

Ticks, which belong to the phylum Arthropoda, are blood-sucking ectoparasitic vectors, characterized by the variety of pathogens they transmit, by their effect on both human and animal health, and by their worldwide socioeconomic involvement. They are divided into two large families: Argasidae (soft ticks) and Ixodidae (hard ticks). Hard ticks are the largest and medically most significant group. It is necessary to assess the distribution of tick-borne pathogens and identify possible risk areas because of the increase in tick-borne disease frequency throughout the world. In Europe, the most common and significant tick is *Ixodes ricinus* (castor bean tick), in terms of its wide ecosystem distribution and the diversity of transmitted pathogens [1].

Among the pathogens transmitted by *I. ricinus*, spirochetes of the *Borrelia burgdorferi sensu lato (s.l.)* complex, the causative agents of human Lyme borreliosis (LB) disease [2] and the Western European TBEV subtype (TBEV-Eur) that causes tick-borne encephalitis (TBE), affect human health to a great degree [3].

*I. ricinus* can also harbor and transmit bacteria of the order Rickettsiales that is important in terms of medical and veterinary concern. Rickettsiae of the spotted fever group (SFG) (*R. helvetica*, *R. monacenis*) lead to rickettsioses in humans [4], *Anaplasma phagocytophilum* causes granulocytic anaplasmosis in both humans and animals [5], while the emerging pathogen “*Candidatus Neoehrlichia mikurensis*” may lead to neoehrlichiosis, primarily a disease of immunocompromised patients [6]. Additionally, *I. ricinus* can also harbor and transmit protozoans of the genus *Babesia*, mainly *B. microti* and *B. divergens*, causative agents of babesiosis in humans, while *B. venatorum* pathogenicity to humans is under investigation [7]. The role of *I. ricinus* in transmission of *Bartonella* species (e.g., *B. henselae* and *B. quintana*), which cause bartonellosis in humans, is discussed controversially [8]. The Q fever agent *Coxiella burnetii* and *Francisella tularensis*, which are causative agents of tularemia, have also been detected in *I. ricinus* [9].

The ixodid ticks undergo the three-host life cycle, whereby the tick leaves the host after the larval and nymphal stages. Moreover, they stay attached to their hosts for up to several days while feeding [10]. The life cycles and transmission of the most infectious agents of ticks are inextricably linked to climate. Climate change has already impacted the transmission of a wide range of vector-borne diseases in Europe [11]. In fact, the ozone hole, the greenhouse effect, the heat waves, the extreme weather events, changes in rainfall patterns, and biodiversity reduction affect the spatial spread of vector-borne diseases [12,13]. Furthermore, climate change indirectly influences the ecosystems by redefining the distribution of the fauna. As a result, global warming is one of the most important factors in redistribution of the ticks’ population and the outbreak of tick-borne diseases [14,15]. In addition, climate change influences the population ecology of *Ixodes* ticks via its effects on abiotic factors such as vegetation, drought, and humidity. This is reflected in the earlier vegetation onset and the extension of the growing season during the year, the levels of drought and humidity, as well as the potential access to water, which is vital for arthropod vectors [16]. 

During the last decades, the prevalence of tick-borne diseases has increased in Europe, due to several biotic and abiotic factors [17,18]. In fact, while in the past tick-borne diseases were endemic only in some specific regions, currently they have been redistributed on an impressively larger geographical scale [19]. TBE and LB disease are considered the most climate-sensitive diseases. In fact, a systematic literature review from 1997 to 2017 revealed that the most climate-sensitive diseases are transmitted by arthropods (51% of diseases that are sensitive to climatic factors), in which 41% belong to tick-borne diseases [16]. 

Discovering which abiotic factors have an impact on the biology of ticks, as well as evaluating seasonal patterns of activity resulting from several combinations of climatic conditions, could constitute an estimation of “risk” in a climate change scenario [20]. Statistical models are useful in order to predict tick distributions by linking their existence to environmental conditions. Therefore, the use of climate and environmental data can help in making spatial predictions of disease distribution [21,22].

Τhe purpose of this review is to present the data concerning the effect of the climatic factors on the prevalence of ticks in Europe and the contribution of climate models as a risk assessment approach for the most prevalent tick-borne diseases. Climate models could predict the redistribution of the *I. ricinus* population and, as a result, they can reveal which areas are more prone to tick-borne diseases. Although accurate prediction of future epidemiological data is not possible, by studying long-term climate changes in the seasons that affect the prevalence and the epidemiology of the ticks, one could potentially minimize the risk of transmission of tick-borne diseases [22].

## 2. How Climate Affects Tick Phenology—The Example of *I. ricinus*

*I. ricinus* is widely distributed across Europe. Its existence has been recorded in countries such as the UK, Germany, Sweden, Ukraine, Russia, Italy, France, Spain, Romania, Belgium, Austria, Latvia, and Lithuania [20,23,24,25,26,27,28,29,30,31,32,33,34]. It is also found in Greece and particularly in Northwestern Greece and Crete, according to the current ECDC map distribution for Europe in March 2021 [35,36].

*I. ricinus* seasonal population dynamics are influenced by both abiotic (climatic) factors acting on the free-living tick stages and biotic (host) responses to the tick as a parasite [37]. Also, interaction between abiotic and biotic factors caused by the indirect effects of climate change on the hosts, influence the abundance and the distribution of the tick [38].

Since the *I. ricinus* species complex spends most of its life (~98%) in the external environment, their prevalence is affected by microclimate changes such as temperature, saturation deficit, humidity, and day length (diapause) [37,39]. High temperature results in a numerical increase of ticks and the extension of the interaction time between ticks and humans [40,41]. Combined with temperature, humidity is an important limiting factor in *I. ricinus* survival and activity. Consequently, relative humidity needs to be above 70–80% in order to allow questing tick behavior and survival [42]. 

Τhe initiation and cessation of questing activity of *I. ricinus* is strongly correlated to the temperature. *I. ricinus*, in order to start questing, requires a relative humidity over 45% and a temperature of 7–8 °C [43]. Observations made mainly in Central Europe revealed that *I. ricinus* ticks are inactive during the winter (mid-November to mid-February), while they actively look for hosts from March to October. In autumn, nymphs and adult *I. ricinus* ticks are more sensitive to a temperature drop, entering behavioral diapause at near-ground temperatures greater than those associated with the onset of questing behavior in the spring period [44]. Questing is performed when temperatures exceed 5 °C [15,44], a theory which has been proved experimentally with the research of Gilbert, Aungier, and Tomkins, 2014, in which ticks of the species *I. ricinus* proceeded to questing in vitro only if the temperature was higher than 6 °C [45].

It is noteworthy, however, that for ticks which were collected from colder climate areas, questing start temperatures were lower than the required temperatures for ticks coming from warmer climate areas. This was also observed for the required temperatures of the tick’s metabolic initiation [46,47]. The lowest temperatures that permit the metabolic function of *I. ricinus* are between −5 °C and −10 °C. These temperatures are probably close to the temperatures that permit the tick’s survival [48].

Many studies reveal that *I. ricinus* can adapt to climate changes and integrate to local climate conditions [45]. In the publication of Gray et al., 2009, it is stated that the questing period in the UK is approximately 10 months per year because of the milder winter climatic conditions in Western Europe. Temperature increases in colder areas affect the winter activity of ticks, leading to the indication that an increase in the activity of *I. ricinus* throughout the year is induced by global warming. The rising winter temperatures are considered to be a critical factor for host survival [49]. On the other hand, an environment that combines a low rate of summer rainfall with summer drought is probably an inhibitor of the growth of *I. ricinus* because it weakens its activity [39].

Low temperatures have a negative effect on the tick’s lipids and water levels, resulting in a later embryogenesis [50]. The way that temperature affects the tick’s metabolism needs to be further investigated in order to clarify the potential tick’s population, their phenology, their seasonal behavior, and the transmission of tick-borne pathogens [39,51]. The results of Alasmari and Wall, 2021, in their study of the temperature’s impact on the metabolic rate and resource depletion for the tick *I. ricinus*, showed a low rate of reduction in the nymphs’ proteins, sometimes also in low temperatures [48].

Climate change influences the latitudinal and altitudinal distribution of *I. ricinus* [38] (Table 1). In Central Sweden, the increase in the population of species is attributed to warmer winters, since today there are more days with temperatures above 7 °C [52,53]. This expansion is attributed to climate change that has caused the northern spread of fauna and flora in Sweden over the last 30 years and includes the upward (on latitude) extension of *I. ricinus* [54]. In the past, the highest latitude with a presence of *I. ricinus* was 61° N [53]. Today, *I. ricinus* is located at latitudes up to 66° N, entirely occupying the Baltic Sea coastline, northern river valleys, and large northern lakes. The reduction of exposure to temperatures below −12 °C for long periods in northern areas is the main parameter that allowed the establishment of ticks. The above is confirmed by the fact that this tick can survive after 24 h exposure in the extreme temperature range of −14.4 °C to −18.9 °C. Instead, an exposure of one month at −10 °C is mostly fatal for the unfed nymphs and for the larvae and nymphs in diapause [55]. Moreover, if *I. ricinus* is not fully developed before the winter, where growth is impossible in the range 7–10 °C, it is impossible for it to survive [39].

Furthermore, the increasing prevalence of spring and autumn conditions results in the expansion of the days with temperatures higher than 5–8 °C. In Central and Western Sweden, the duration of tick activity is 6 to 8 months per year, whereas for the British Isles, this period is counted as 11 months per year or the whole year [39].

At higher altitudes, without optimal temperature, ticks have less time to search for a host. Consequently, the chances of survival are less compared with ticks at lower altitudes [56]. The altitude has a negative correlation with the biological integration of ticks, due to the decrease in temperature that limits the period of questing and development [37,47,57]. Low temperatures in spring delay the ticks’ activation of the questing [47] as a result of altitude variation [57]. Climate change is linked with the increase in tick populations at higher altitudes in Czech Republic (Table 1) and in Scotland, where the mean temperature is increasing [56]. 

Apart from temperature, saturation deficit, indicator of air dryness that depends on temperature and relative humidity, is an important factor influencing the tick population [56]. Ιt influences desiccation, and combined with the day length, it affects the relationship of the tick’s diapause, which is defined as the metabolic and developmental hold-up of the tick, caused by environmental changes. Moreover, it is considered to be an essential factor for questing [58]. For example, in Sweden, fewer ticks are observed in areas with low altitude and large saturation deficit [56].

**Table 1:** Current and future state of the effect of climate change on *I. ricinus* prevalence and on tick-borne diseases in some European countries.

	Country	Changes in *I. ricinus* Prevalence	Current Incidence of Tick-Borne Diseases	Future Incidence of Tick-Borne Diseases (by 2050)	Ref.
Northern Europe	Czech Republic	Has expanded into higher altitudes as a response to increases in average temperatures	Ιncreasing incidence of TBE	Ιncreasing incidence of TBE	[59]
	Sweden	Longer tick activity seasons Distribution-limit shifted to higher latitude	Ιncreasing incidence of LB	Ιncreasing incidence of tick-borne disease in southern parts of the country	[60]
	Norway	Distribution-limit shifted to higher latitude	Decreasing incidence of TBE	Ιncreasing incidence of tick-borne disease in southern parts of the country	[60]
	Finland	Amplifying tick questing activity Prolonging the duration of the tick activity season Distribution-limit shifted to higher altitudes and latitudes	Ιncreasing incidence of LB	Ιncreasing incidence of tick-borne disease in southern parts of the country	[60]
	Germany	Distribution-limit shifted to higher latitude	Ιncreasing incidence of LB	Ιncreasing incidence of LB	[61]
Southern Europe	Greece	Has expanded to include areas at higher latitude and/or altitudes	Low incidence of tick-borne diseases	Have reduced areas of high- risk incidence of tick-borne diseases	[60]
	Italy	Has expanded to include areas at higher latitude and/or altitudes	Low incidence of tick-borne diseases	High-risk incidence of tick-borne diseases	[60]
	Portugal	Has expanded to include areas at higher latitude and/or altitudes	Low incidence of tick-borne diseases	Have reduced areas of high- risk incidence of tick-borne diseases	[60]

## 3. The Effect of Climate Change on the Prevalence of LB and TBE

Climate change plays a causative role in the outbreak of tick-borne diseases (Table 1). Τhe impact of climate change on seasonal outbreaks or recessions of diseases transmitted by vectors in Europe has been described in many studies [16,22,62]. It is characteristic that the increase in temperature causes a significant reduction in the time required for incubation of the pathogen and for the vector’s life cycle, while the risk of the transmission to humans is dramatically increased due to the increase of the vector’s population [22].

LB is the most commonly transmitted to humans tick-borne disease in Europe and *I. ricinus* is the principal tick vector in Europe [63].The abundance of the infected *I. ricinus* nymphs is the best determinant factor that defines the transmission risk to humans and is influenced by the reduction in the altitude, due to the increase of the temperature [64]. The change in the hosts and in the habitat is likely to provoke an increase of *I. ricinus* density and the transmission risk of LB in Northwestern Europe, which is characterized by cold temperate climate and also by more elevated altitudes [38].

Humidity, temperature, and saturation deficit are the most important abiotic factors affecting LB vectors [56]. The consequences of global climate change converge on the fact that LB is going to remain a major health issue for the future [65]. The risk of LB transmission is connected with the increase of the hibernal and summery temperatures and the small alternations in the intraseasonal temperature range [66]. Moreover, the highest possibility of infection happens throughout the warmest period of the year, from May until September, with a peak in July [65].

The increase in the prevalence of the disease in Europe is noticeable and is attributed synergistically to anthropogenic and climatic factors [38]. In the decades 1990–2010, more than 360,000 cases were recorded in Europe [67], which represents a 400% increase of the prevalence of LB in endemic regions [67,68]. Currently, every year, there are estimated 65,000 new cases of the disease [22]. However, this number constitutes only an estimation, due to the incomplete recording of the cases in all the European countries [65]. 

In Western Europe, there are recorded 22.05 LB cases per 100,000 people per year [69]. Lithuania has one of the highest prevalence rates of the disease per country in Europe, resulting in mandatory registry of the disease in this country. For the years 2011–2018, there were recorded 73.9–100.6 cases per 100,000 people, while from 2014 to 2016, there were recorded 7424 new cases, which is equivalent to an approximate incidence rate of 85.4 [65]. The incidence rate is lower for the countries of Northern and Southwest Europe, which are characterized by the lowest and highest temperatures in Europe, respectively [70] (Table 2).

Another tick-borne disease that is affected by climate change is TBE. Study of its prevalence is of utmost importance since it is considered one of the most serious neurological diseases transmitted by tick bites in Central Europe, Eastern Europe, and Russia, and since it has a significant impact on public health in these geographical regions. TBEV is transmitted by 11 tick species, but only 2 tick species are important vectors of TBEV: *I. ricinus* and *I. persulcatus* [78]. The environmental conditions required for the transmission of the homonymous Flavivirus TBEV include high rates of humidity. In these conditions, the species *I. ricinus* can survive for a long time, actively looking for hosts. Moreover, the daily temperature (land surface temperature—LST) determines the epidemiology of TBE and how it is distributed seasonally [18]. A temperate climate is usually necessary for the transmission of TBE [79]. Τhe warm weather is responsible for the increase of the prevalence of TBE [80]. An increase in the number of the *I. ricinus* ticks is predicted due to the rise of the temperature, which is also going to accelerate the maturation of the ticks [81]. The temperature rise constitutes a risk of increased TBE cases from a possible transmission, which occurs during winter, as it causes the extension of the tick’s activity period during the winter months and consequently the reduction of the tick’s inactivity period. Moreover, the higher temperatures that occur as a result of climate change influence the length of the round fluctuations in TBEV in numerous European countries such as Sweden, Germany, Czech Republic, Slovenia, Austria, and Italy [82]. Τhe simultaneous blood meal of *I. ricinus* uninfected larvae and infected nymphs from a host correlates with the occurrence of TBE cases and depends on the rhythm of the temperature reduction in the autumn [21]. A premise for the presence of TBEV is the greatest rate of the temperature’s fall in the autumn months compared to the average of higher summer temperatures. Climate change might affect the method of transmission of TBE more largely than the geographical localization of *I. ricinus* [83]. The above can be explained by the shift of the seasonal coincidence of the nymphs and larvae simultaneously fed from the same host, which alters the path of transmission [83]. 

The emergence of TBE in Estonia, Latvia, and Lithuania cannot be attributed mainly to climate, but is more likely to be attributed to the combined action of climate change which coincided with the conditions that were created by the dissolution of the Soviet Union [84], such as the collapse of the Health and Welfare system. On the contrary, the expected increase of the seasonal activity of *I. ricinus* due to climate change is reflected in the emergence of TBE during a period of almost 30 years (1960–1998) in Stockholm, Sweden [85]. 

The increased prevalence of TBE in Sweden, an endemic region of the disease, was associated in the 1980s with two consecutive years of less winter cold, earlier spring and later autumn, with temperatures above 5–8 °C [85]. The spread of *I. ricinus* in northern latitudes and at higher altitudes in Sweden demonstrates the contribution of climate as a cause of increasing prevalence of TBE disease, similar to the emergence of LB [52,54].

The disease is endemic in Czech Republic and has been recorded since 1971 in a special database. This database collects data on TBE cases that were present in the country. The weekly recording of the incidents, depending on the weather conditions and the presence of ticks, had already been started in 1954, with the creation of bimodal distributions per week. That was the year when the influence of the weather conditions was recognized [86]. Since then, 21,847 cases have been recorded over a period of 36 years (up to 2016), enabling the epidemiological study of TBE as a function of climate change [86].

The most important variable for the questing forecast of the TBE vector is the temperature [86]. Specifically, the air temperature prevails as the best factor for the prediction of the questing, compared to other factors such as the photoperiod and the humidity [44]. The conclusion agrees with the research of [87], which studies the impact of the disease in relation to temperature and rainfall. This is indicated by the fact that fluctuations in tick-borne activity and the incidence of the disease in the two seasonal periods are a result of the fact that more TBE infections occur in higher temperatures. In addition, fewer infections are caused in the spring, rather than the summer season, despite the fact that in the summer there is an explosive increase in the nymphs’ search for hosts and the fact that people start going out in nature. This is explained by the fact that the lower spring temperatures can be an inhibitory factor for virus replication, especially after low winter temperatures, so that the minimum viral load that is needed for the virus transmission, and for the clinical manifestation of the disease that is caused, is not reached. Unlike the protection that the spring offers due to the lower temperatures, the higher temperatures during the summer–autumn period may increase the risk of transmission of TBE after a possible tick bite [86].

It is also noted that the average weekly temperature can be a better risk factor for the prediction of ΤΒΕ, in comparison with the daily air temperature. At the same time, research in Central Bohemia and in various parts of the Czech Republic prove the effect of the near-ground temperature as the most important study factor for tick activity [44,88].

Furthermore, concerning the factors that influence the prevalence of TBE, there isa correlation between extreme weather events and the behavior of ticks [86]. Τhis was shown by the reverse of the slope in the correlation model between the number of cases of TBE and the number of active nymphs for the year 2003 in Czech Republic. This reverse was attributed to the unprecedented events of the year, such as the high drought that was noticed from March to September, 2003, which caused an average increase in temperature and intense floods in that year. It is believed that these phenomena are the cause of the unexpected seasonal epidemiological picture of TBE in 2003 [44]. 

In a recognized TBE endemic region in Hungary, climate change is going to increase the co-feeding ticks, which is going to increase the risk of transmission and the prevalence of TBE. For the years 2021–2050 and 2071–2100 31% and 50% increase is estimated, respectively, compared to the period1961–1990. In parallel, it is noted that these results are influenced by the reduction of the forests and the movement of the animals that are hosts of the tick in northern latitudes as a result of climate change and human activities. These factors might result in the reduction of the TBE’s incidence in the enzootic cycle [81]. In Greece, during the last decades, there has been an increase in the risk of TBE. In the past, no TBE cases were observed in the country. Recently, a case was recorded in Eastern Macedonia, in 2014 [89]. Moreover, one case was recorded in 2015 in Peloponnese, and two cases in 2018 and four cases in 2021 in Central Macedonia, Thessalia, and Thrace [90].

The annual incidence of these diseases is also increasing in many countries in Central, Northern, and Eastern Europe and in European areas in which these diseases have never been observed before [91]. This emergence is attributed to economic, social, environmental, and climatic factors [92]. 

## 4. Climate Models as a Useful Tool in Predicting the Risk of Tick-Borne Diseases

Climate change is considered a crucial factor driving the range expansion of arthropod vectors and the increased incidence of vector-borne diseases. Thus, it is important to determine which abiotic and biotic factors are influencing the abundance of *Ixodes* ticks in order to predict the risk of tick-borne diseases [93]. Mathematical models are widely used. They include fundamental biological mechanisms, enabling predictions regarding the relative impact of climate change. However, in most cases, these models make simplifying assumptions regarding vector or pathogen dynamics, without incorporating data regarding the factors that drive the vector-borne diseases, such as seasonality in vector as well as host and pathogen dynamics. Although the current approaches are focused on understanding and predicting how climate change influences the abundance of vectors mainly in existing endemic areas, we do not have a clear view. Moreover, it is necessary to develop and use climatic models that forecast risk in new regions. Consequently, scientists, in order to investigate how climate affects the distribution range of *I. ricinus* species, knowing the possibility of the shift in the distribution of the species due to climate change [38], have to collect data for climate variables that are known to be important for tick ecology (e.g., temperature, relative humidity, saturation deficit, and precipitation). Importantly, climate models that can predict changes in the short and long term have been developed [22,94].

Previous studies used two different kinds of models to forecast future changes in the potential distribution of ticks under different scenarios of environmental change. The first one was the ecological-process-based tick population model and the second one was the associative species distribution model (SDM). The maximum entropy model (MaxEnt) is an SDM method widely used in projecting the spatial distribution of ticks. The MaxEnt model has many advantages because it uses both presence data and categorical data as input variables. In addition, it can be applied in small sample sizes, evaluating the significance level of individual environmental variables using a built-in jackknife test [95].

Theoretical models have predicted that *Ixodes* ticks will increase their distribution and abundance under global warming [96]. In fact, the implementation of the A2 CSIRO STRES climate change scenario has demonstrated a future extension of the tick species to higher altitudes and latitudes, such as in Scandinavia, the Baltic states, and Belarus [97]. At the same time, a reduction of tick populations is expected in the Alps, the Pyrenees, in Central and Western Italy, and in Northwestern Poland. The Scenario A2 for climate change predicts a 3.8% increase in overall spread of the tick species, from 24.2% to 28% in the examined areas. Despite the population limitation in the areas of the Alps, Pyrenees, Italy, Poland, France, Germany, Croatia, and Spain, a probable increase in the Scandinavian countries (Sweden, Norway, Finland), in the countries of the Baltic Sea (Estonia, Latvia, Lithuania), Denmark, and Belarus would be observed.

Presence data and bioclimatic variables were used and a prediction model was created using two scenarios of greenhouse gas emissions and consequent climate change: representative concentration pathway (RCP) 4.5, representing medium-to-low emissions levels, and RCP 8.5, representing the high emissions scenario [98]. For Europe, the application of RCP 4.5 climate scenario proposes a dispersion of 10.8% and 11.5% of *I. ricinus*, and the implementation of RCP 8.5 predicts an increase of the dispersion of the tick of 11.7% and 14.5% by 2050 and 2070, respectively. Overall, the difference in distribution for the year 2050 with the implementation of RCP 4.5 and RCP 8.5 is 0.9%. The same difference for the year 2070 is 1.3%. Beyond the future presence of ticks in areas that are still found today, such as large areas of Western and Central Europe and smaller ones in Northern Europe, a probable expansion of the tick is located in North and East Europe. However, it is noted that the forecasts for the future situation in Mediterranean areas are considered uncertain, as the models that have been implemented cannot guarantee with certainty the geographical distribution there [99]. 

Surprisingly, a recent study suggested that there is no direct association between climate change and the doubling of nymphal tick abundance at three different elevations on Chaumont Mountain in Neuchâtel, Switzerland. However, based on Akaike information criterion (AIC)-based model analysis, it has been found that seed production by beech trees is an ecological variable that drives annual variation in tick density. This finding indicates that climate change could be increasing tick abundance in endemic areas via indirect effects on tree seed production [100]. 

In parallel, another recent study suggests extreme changes of tick dynamics and density, with a pronounced shift of maximum peak activity towards earlier times of the year and a dramatic increase of tick density across Germany. These results were extrapolated after implementation of ecological models with high-resolution climate projection data of temperature, precipitation, and relative humidity for the period 1971–2099 from 15 different climate models. A climate-driven cohort-based population model was used to study tick activity, whereas spatial density changes predictions were based on the extrapolation of a Germany-wide tick density model [101]. 

Today, the few models that study LB are based on temperature changes as the definite factor that can predict the potential elevated risk of its prevalence [72]. However, these findings need to be co-estimated with other factors such as land use and the host population distribution, in order to produce more accurate predictive models [71]. In this regard, a recent study that evaluates the prevalence of LB in Slovenia, which is among the top European countries regarding the number of people infected with LB, showed that climate change triggered a spatial shift of existing foci of LB. The projected potential shifts in LB foci by 2050 and 2070 were calculated after applying five significantly different global climate models (Hadley Center Global Environment Model version 2—Earth System configuration (HadGEM2-ES), The Community Climate System Model 4 (CCSM4), MIROC Earth System Model (MIROCESM), Hadley Center Global Environment Model version 2—low top configuration (HadGEM2-CC), and The Coupled Max Planck Institute Earth System Model at base resolution (MPI-ESM-LR)) according to the RCP 8.5 climate scenario. Data analysis showed that the infection risk could increase by up to 10% by the end of the century in Slovenia, highlighting the need for better preparation of mitigation plans and preventive action implementation [102]. 

The numbers of reported TBE cases in Europe have increased in several endemic regions in recent decades, indicative of an increasing threat to public health. Regarding the impact of climate change on TBE prevalence, mathematical models assess the TBEV transmission dynamics involving both systemic and co-feeding transmission routes in order to predict the transmission risk of TBEV. In Hungary, scientists have provided an effective tool for quantifying the transmission risk of TBE. Based on the prediction that the temperature will increase in 2021–2050 and 2071–2100, they have predicted changes in the ecology of the ticks. For example, there will be an extension of the tick questing season and an increase in the numbers of susceptible ticks (larval and nymphal) and the number of infected nymphal ticks co-feeding on the same hosts, leading to a compounded increase of infections through systemic transmission from then on. These observations result in an enhanced transmission potential and the risk in the study site is expected to increase along with the increase of the temperature [81]. In Finland, scientists used eight predictive modelling techniques: generalized linear models (GLM), generalized additive models (GAM), classification tree analysis (CTA), artificial neural networks (ANNs), multivariate adaptive regression splines (MARS), generalized boosting models (GBM), random forest (RF), and MaxEnt in order to identify the risk areas of TBE transmission and the factors that best explain the presence of human TBE cases. Based on the predictive maps, high-risk areas for TBE transmission were located in the coastal regions in Southern and Western Finland (including the Åland Islands), several municipalities in Central and Eastern Finland, and coastal municipalities in Southern Lapland. To explore potential changes in TBE distributions in the future climate, we used bioclimatic factors with current and future climate forecast data to reveal possible future hotspot areas. Based on the future forecasts, a slightly wider geographical extent of TBE risk was introduced in the Åland Islands and Southern, Western, and Northern Finland, even though the risk itself was not increased [103].The studies described above rely heavily on the changes in climatic variables, i.e., temperature, precipitation, etc., and do not incorporate data regarding the land use/cover change (LUCC) that is known to affect the geographic distribution of tick habitats. LUCC can change not only the local surface conditions but also microclimatic conditions, which could affect habitat suitability for ticks and their host animals to various extents. 

## 5. Conclusions

Climate change has a great impact on the transmission of tick-borne diseases, by affecting the interactions of the tick–host–pathogen triangle. Both the increase of the temperature and changes in the rainfall patterns affect the tick’s ability to survive, evolve, and function. Although models of future spatial distribution of ticks in European countries have been extensively reviewed, the measures taken to ensure the epidemiological treatment to date are limited. The need for epidemiological surveillance is considered to be of major importance since the tick-borne diseases, influenced by climate change, do not only constitute a future threat, but at the same time affect the present. 

## Figures and Tables

**Table 2:** Incidence rate for LB in Europe.

Region	Incidence Rate (100,000 People per Year)	Reference
Western Europe	22.05 cases	[69]
France (2009–2017)	53 cases	[71]
Northern Italy (2000–2015)	12.4 cases	[72]
United Kingdom	12.1 cases	[73]
Finland	61 cases	[74]
Iceland	2 cases	[75]
Spain	2.5–11.6 cases	[76]
Lithuania	99.9 cases	[65]
Germany	400 cases	[77]
Regions of Slovenia, Austria, Baltic Coastline of Northern Sweden, some Estonian and Finnish Islands	100 cases	[2]

Νote: the collection of data concerns either an annual rate or a rate for a period of years when this time is mentioned.

## Data Availability

Publicly available datasets were analyzed in this study.

## References

[1] Černý J., Lynn G., Hrnková J., Golovchenko M., Rudenko N., Grubhoffer L. (2020). Management Options for Ixodes ricinus-Associated Pathogens: A Review of Prevention Strategies. Int. J. Environ. Res. Public Health.

[2] Rizzoli A., Hauffe H.C., Carpi G., Vourc’h G.I., Neteler M., Rosà R. (2011). Lyme borreliosis in Europe. Eurosurveillance.

[3] Gritsun T.S., Lashkevich V.A., Gould E.A. (2003). Tick-borne encephalitis. Antiviral Res..

[4] Parola P., Paddock C.D., Socolovschi C., Labruna M.B., Mediannikov O., Kernif T., Abdad M.Y., Stenos J., Bitam I., Fournier P.E. (2013). Update on tick-borne rickettsioses around the world: A geographic approach. Clin. Microbiol. Rev..

[5] Stuen S., Granquist E.G., Silaghi C. (2013). Anaplasma phagocytophilum-a widespread multi-host pathogen with highly adaptive strategies. Front. Cell. Infect. Microbiol..

[6] Grankvist A., Andersson P.O., Mattsson M., Sender M., Vaht K., Höper L., Sakiniene E., Trysberg E., Stenson M., Fehr J. (2014). Infections with the tick-borne bacterium “Candidatus Neoehrlichia mikurensis” mimic noninfectious conditions in patients with B cell malignancies or autoimmune diseases. Clin. Infect. Dis..

[7] Lejal E., Marsot M., Chalvet-Monfray K., Cosson J.F., Moutailler S., Vayssier-Taussat M., Pollet T. (2019). A three-years assessment of Ixodes ricinus-borne pathogens in a French peri-urban forest. Parasites Vectors.

[8] Regier Y., Ballhorn W., Kempf V.A.J. (2017). Molecular detection of Bartonella henselae in 11 Ixodes ricinus ticks extracted from a single cat. Parasites Vectors.

[9] Körner S., Makert G.R., Ulbert S., Pfeffer M., Mertens-Scholz K. (2021). The Prevalence of Coxiella burnetii in Hard Ticks in Europe and Their Role in Q Fever Transmission Revisited—A Systematic Review. Front. Vet. Sci..

[10] Bente D.A., Forrester N.L., Watts D.M., McAuley A.J., Whitehouse C.A., Bray M. (2013). Crimean-Congo hemorrhagic fever: History, epidemiology, pathogenesis, clinical syndrome and genetic diversity. Antiviral Res..

[11] Patz J.A., Campbell-Lendrum D., Holloway T., Foley J.A. (2005). Impact of regional climate change on human health. Nature.

[12] Bittner M.I., Matthies E.F., Dalbokova D., Menne B. (2014). Are European countries prepared for the next big heat-wave?. Eur. J. Public Health.

[13] Thuiller W., Lavorel S., Araújo M.B., Sykes M.T., Prentice I.C. (2005). Climate change threats to plant diversity in Europe. Proc. Natl. Acad. Sci. USA.

[14] Porretta D., Mastrantonio V., Amendolia S., Gaiarsa S., Epis S., Genchi C., Bandi C., Otranto D., Urbanelli S. (2013). Effects of global changes on the climatic niche of the tick Ixodes ricinus inferred by species distribution modelling. Parasites Vectors.

[15] Sprong H., Hofhuis A., Gassner F., Takken W., Jacobs F., Van Vliet A.J.H., Van Ballegooijen M., Van Der Giessen J., Takumi K. (2012). Circumstantial evidence for an increase in the total number and activity of borrelia-infected ixodes ricinus in the Netherlands. Parasites Vectors.

[16] Omazic A., Bylund H., Boqvist S., Högberg A., Björkman C., Tryland M., Evengård B., Koch A., Berggren C., Malogolovkin A. (2019). Identifying climate-sensitive infectious diseases in animals and humans in Northern regions. Acta Vet. Scand..

[17] Pfäffle M., Littwin N., Muders S.V., Petney T.N. (2013). The ecology of tick-borne diseases. Int. J. Parasitol..

[18] Randolph S.E. (2000). Ticks and tick-borne disease systems in space and from space. Adv. Parasitol..

[19] Capelli G., Ravagnan S., Montarsi F., Ciocchetta S., Cazzin S., Porcellato E., Babiker A.M., Cassini R., Salviato A., Cattoli G. (2012). Occurrence and identification of risk areas of Ixodes ricinus-borne pathogens: A cost-effectiveness analysis in north-eastern Italy. Parasites Vectors.

[20] Mannelli A., Bertolotti L., Gern L., Gray J. (2012). Ecology of Borrelia burgdorferi sensu lato in Europe: Transmission dynamics in multi-host systems, influence of molecular processes and effects of climate change. FEMS Microbiol. Rev..

[21] Randolph S.E., Green R.M., Peacey M.F., Rogers D.J. (2000). Seasonal synchrony: The key to tick-borne encephalitis foci identified by satellite data. Parasitology.

[22] Semenza J.C., Suk J.E. (2018). Vector-borne diseases and climate change: A European perspective. FEMS Microbiol. Lett..

[23] Cull B., Pietzsch M.E., Hansford K.M., Gillingham E.L., Medlock J.M. (2018). Surveillance of British ticks: An overview of species records, host associations, and new records of Ixodes ricinus distribution. Ticks Tick-Borne Dis..

[24] Jaenson T.G.T., Jaenson D.G.E., Eisen L., Petersson E., Lindgren E. (2012). Changes in the geographical distribution and abundance of the tick Ixodes ricinus during the past 30 years in Sweden. Parasites Vectors.

[25] Cafiso A., Olivieri E., Floriano A.M., Chiappa G., Serra V., Sassera D., Bazzocchi C. (2021). Investigation of tick-borne pathogens in ixodes ricinus in a peri-urban park in lombardy (Italy) reveals the presence of emerging pathogens. Pathogens.

[26] Akl T., Bourgoin G., Souq M.L., Appolinaire J., Poirel M.T., Gibert P., Abi Rizk G., Garel M., Zenner L. (2019). Detection of tick-borne pathogens in questing Ixodes ricinus in the French Pyrenees and first identification of Rickettsia monacensis in France. Parasite.

[27] Remesar S., Fernández P.D., Venzal J.M., Pérez-Creo A., Prieto A., Estrada-Peña A., López C.M., Panadero R., Fernández G., Díez-Baños P. (2019). Tick species diversity and population dynamics of Ixodes ricinus in Galicia (north-western Spain). Ticks Tick-Borne Dis..

[28] Ben I., Lozynskyi I. (2019). Prevalence of Anaplasma phagocytophilum in Ixodes ricinus and Dermacentor reticulatus and Coinfection with Borrelia burgdorferi and Tick-Borne Encephalitis Virus in Western Ukraine. Vector Borne Zoonotic Dis..

[29] Zając Z., Kulisz J., Bartosik K., Woźniak A., Dzierżak M., Khan A. (2021). Environmental determinants of the occurrence and activity of Ixodes ricinus ticks and the prevalence of tick-borne diseases in eastern Poland. Sci. Rep..

[30] Kalmár Z., Dumitrache M., D’Amico G., Matei I.A., Ionică A.M., Gherman C.M., Lupșe M., Mihalca A.D. (2020). Multiple Tick-Borne Pathogens in Ixodes ricinus Ticks Collected from Humans in Romania. Pathogens.

[31] Rousseau R., Vanwambeke S.O., Boland C., Mori M. (2021). The Isolation of Culturable Bacteria in Ixodes ricinus Ticks of a Belgian Peri-Urban Forest Uncovers Opportunistic Bacteria Potentially Important for Public Health. Int. J. Environ. Res. Public Health.

[32] Capligina V., Seleznova M., Akopjana S., Freimane L., Lazovska M., Krumins R., Kivrane A., Namina A., Aleinikova D., Kimsis J. (2020). Large-scale countrywide screening for tick-borne pathogens in field-collected ticks in Latvia during 2017–2019. Parasites Vectors.

[33] Sidorenko M., Radzijevskaja J., Mickevičius S., Bratčikovienė N., Paulauskas A. (2021). Prevalence of tick-borne encephalitis virus in questing Dermacentor reticulatus and Ixodes ricinus ticks in Lithuania. Ticks Tick-Borne Dis..

[34] Vogelgesang J.R., Walter M., Kahl O., Rubel F., Brugger K. (2020). Long-term monitoring of the seasonal density of questing ixodid ticks in Vienna (Austria): Setup and first results. Exp. Appl. Acarol..

[35] Ixodes Ricinus—Current Known Distribution: March 2021. https://www.ecdc.europa.eu/en/publications-data/ixodes-ricinus-current-known-distribution-march-2021.

[36] Efstratiou A., Karanis G., Karanis P. (2021). Tick-Borne Pathogens and Diseases in Greece. Microorganisms.

[37] Randolph S.E., Randolph S., Randolph S.E. (2004). IJNM Mini-Review Evidence that climate change has caused “emergence” of tick-borne diseases in Europe? Introduction -the undeniable impact of climate on tick-borne diseases. Int. J. Med. Microbiol..

[38] Gilbert L. (2021). The Impacts of Climate Change on Ticks and Tick-Borne Disease Risk. Annu. Rev. Entomol..

[39] Gray J.S., Dautel H., Estrada-Peña A., Kahl O., Lindgren E. (2009). Effects of Climate Change on Ticks and Tick-Borne Diseases in Europe. Interdiscip. Perspect. Infect. Dis..

[40] Alonso-Carné J., García-Martín A., Estrada-Peña A. (2016). Modelling the Phenological Relationships of Questing Immature Ixodes Ricinus (Ixodidae) Using Temperature and NDVI Data. Zoonoses Public Health.

[41] Cat J., Beugnet F., Hoch T., Jongejan F., Prangé A., Chalvet-Monfray K. (2017). Influence of the spatial heterogeneity in tick abundance in the modeling of the seasonal activity of Ixodes ricinus nymphs in Western Europe. Exp. Appl. Acarol..

[42] Hauser G., Rais O., Morán Cadenas F., Gonseth Y., Bouzelboudjen M., Gern L. (2018). Influence of climatic factors on Ixodes ricinus nymph abundance and phenology over a long-term monthly observation in Switzerland (2000–2014). Parasites Vectors.

[43] Hubálek Z., Halouzka J., Juricová Z. (2003). Host-seeking activity of ixodid ticks in relation to weather variables. J. Vector Ecol..

[44] Daniel M., Malý M., Danielová V., Kəíž B., Nuttall P. (2015). Abiotic predictors and annual seasonal dynamics of Ixodes ricinus, the major disease vector of Central Europe. Parasites Vectors.

[45] Gilbert L., Aungier J., Tomkins J.L. (2014). Climate of origin affects tick (Ixodes ricinus) host-seeking behavior in response to temperature: Implications for resilience to climate change?. Ecol. Evol..

[46] Tomkins J.L., Aungier J., Hazel W., Gilbert L. (2014). Towards an evolutionary understanding of questing behaviour in the tick Ixodes ricinus. PLoS ONE.

[47] Perret J.L., Guigoz E., Rais O., Gern L. (2000). Influence of saturation deficit and temperature on Ixodes ricinus tick questing activity in a Lyme borreliosis-endemic area (Switzerland). Parasitol. Res..

[48] Alasmari S., Wall R. (2021). Metabolic rate and resource depletion in the tick Ixodes ricinus in response to temperature. Exp. Appl. Acarol..

[49] Pecl G.T., Araújo M.B., Bell J.D., Blanchard J., Bonebrake T.C., Chen I.C., Clark T.D., Colwell R.K., Danielsen F., Evengård B. (2017). Biodiversity redistribution under climate change: Impacts on ecosystems and human well-being. Science.

[50] Daniel M. (1993). Influence of the microclimate on the vertical distribution of the tick Ixodes ricinus (L) in Central Europe. Acarologia.

[51] Burtis J.C., Sullivan P., Levi T., Oggenfuss K., Fahey T.J., Ostfeld R.S. (2016). The impact of temperature and precipitation on blacklegged tick activity and lyme disease incidence in endemic and emerging regions. Parasites Vectors.

[52] Lindgren E., Tälleklint L., Polfeldt T. (2000). Impact of climatic change on the northern latitude limit and population density of the disease-transmitting European tick Ixodes ricinus. Environ. Health Perspect..

[53] Jaenson T.G.T., Talleklint L., Lundqvist L., Olsen B., Chirico J., Mejlon H. (1994). Geographical distribution, host associations, and vector roles of ticks (Acari: Ixodidae, Argasidae) in Sweden. J. Med. Entomol..

[54] Tälleklint L., Jaenson T.G.T. (1998). Increasing Geographical Distribution and Density of Ixodes ricinus (Acari: Ixodidae) in Central and Northern Sweden. J. Med. Entomol..

[55] Dautel H., Knülle W. (1997). Cold hardiness, supercooling ability and causes of low-temperature mortality in the soft tick, Argas reflexus, and the hard tick, Ixodes ricinus (Acari: Ixodoidea) from Central Europe. J. Insect Physiol..

[56] Gilbert L. (2010). Altitudinal patterns of tick and host abundance: A potential role for climate change in regulating tick-borne diseases?. Oecologia.

[57] Jouda F., Perret J.L., Gern L. (2004). Ixodes ricinus density, and distribution and prevalence of Borrelia burgdorferi sensu lato infection along an altitudinal gradient. J. Med. Entomol..

[58] Needham G.R., Teel P.D. (1991). Off-host physiological ecology of ixodid ticks. Annu. Rev. Entomol..

[59] Comparison of the Epidemiological Patterns of Lyme Borreliosis and Tick-Borne Encephalitis in the Czech Republic in 2007–2016—PubMed. https://pubmed.ncbi.nlm.nih.gov/30602281/.

[60] Li S., Gilbert L., Vanwambeke S.O., Yu J., Purse B.V., Harrison P.A. (2019). lyme disease risks in europe under multiple uncertain drivers of change. Environ. Health Perspect..

[61] Enkelmann J., Böhmer M., Fingerle V., Siffczyk C., Werber D., Littmann M., Merbecks S.S., Helmeke C., Schroeder S., Hell S. (2018). Incidence of notified Lyme borreliosis in Germany, 2013–2017. Sci. Rep..

[62] Semenza J.C., Menne B. (2009). Climate change and infectious diseases in Europe. Lancet Infect. Dis..

[63] Kullberg B.J., Vrijmoeth H.D., Van De Schoor F., Hovius J.W. (2020). Lyme borreliosis: Diagnosis and management. BMJ.

[64] James M.C., Bowman A.S., Forbes K.J., Lewis F., McLeod J.E., Gilbert L. (2013). Environmental determinants of Ixodes ricinus ticks and the incidence of Borrelia burgdorferi sensu lato, the agent of Lyme borreliosis, in Scotland. Parasitology.

[65] Petrulionienė A., Radzišauskienė D., Ambrozaitis A., Čaplinskas S., Paulauskas A., Venalis A. (2020). Epidemiology of LB in a highly endemic European zone. Medicina.

[66] Estrada-Peña A., Ortega C., Sánchez N., DeSimone L., Sudre B., Suk J.E., Semenza J.C. (2011). Correlation of Borrelia burgdorferi Sensu lato prevalence in questing Ixodes ricinus ticks with specific abiotic traits in the Western Palearctic. Appl. Environ. Microbiol..

[67] Marrama-Rakotoarivony L., Sudre B., Bortel W.V., Warns-Petit E., Zeller H. (2014). Annual Epidemiological Report Emerging and Vector-Borne Diseases.

[68] Medlock J.M., Hansford K.M., Bormane A., Derdakova M., Estrada-Peña A., George J.C., Golovljova I., Jaenson T.G.T., Jensen J.K., Jensen P.M. (2013). Driving forces for changes in geographical distribution of Ixodes ricinus ticks in Europe. Parasites Vectors.

[69] Sykes R.A., Makiello P. (2017). An estimate of Lyme borreliosis incidence inWestern Europe. J. Public Health.

[70] Jore S., Viljugrein H., Hofshagen M., Brun-Hansen H., Kristoffersen A.B., Nygård K., Brun E., Ottesen P., Sævik B.K., Ytrehus B. (2011). Multi-source analysis reveals latitudinal and altitudinal shifts in range of Ixodes ricinus at its northern distribution limit. Parasites Vectors.

[71] Gocko X., Lenormand C., Lemogne C., Bouiller K., Gehanno J.F., Rabaud C., Perrot S., Eldin C., de Broucker T., Roblot F. (2019). Lyme borreliosis and other tick-borne diseases. Guidelines from the French scientific societies. Med. Mal. Infect..

[72] Zanzani S.A., Rimoldi S.G., Manfredi M.T., Grande R., Gazzonis A.L., Merli S., Olivieri E., Giacomet V., Antinori S., Cislaghi G. (2019). Lyme borreliosis incidence in Lombardy, Italy (2000–2015): Spatiotemporal analysis and environmental risk factors. Ticks Tick-Borne Dis..

[73] Cairns V., Wallenhorst C., Rietbrock S., Martinez C. (2019). Incidence of lyme disease in the UK: A population-based cohort study. BMJ Open.

[74] Sajanti E., Virtanen M., Helve O., Kuusi M., Lyytikäinen O., Hytönen J., Sane J. (2017). Lyme borreliosis in Finland, 1995–2014. Emerg. Infect. Dis..

[75] Vigfússon H.B., Haroarson H.S., Lúovíksson B.R., Guolaugsson Ó. (2019). LB in Iceland—Epidemiology from 2011 to 2015. Laeknabladid.

[76] Vázquez-López M.E., Pego-Reigosa R., Díez-Morrondo C., Castro-Gago M., Díaz P., Fernández G., Morrondo P. (2015). Epidemiology of LB in a healthcare area in north-west Spain. Gac. Sanit..

[77] WHO (2012). Accelerating Work to Overcome the Global Impact of Neglected Tropical Diseases: A Roadmap for Implementation.

[78] Amicizia D., Domnich A., Panatto D., Lai P.L., Cristina M.L., Avio U., Gasparini R. (2013). Epidemiology of tick-borne encephalitis (TBE) in Europe and its prevention by available vaccines. Hum. Vaccines Immunother..

[79] Petri E., Gniel D., Zent O. (2010). Tick-borne encephalitis (TBE) trends in epidemiology and current and future management. Travel Med. Infect. Dis..

[80] Nah K., Magpantay F.M.G., Bede-Fazekas Á., Röst G., Trájer A.J., Wu X., Zhang X., Wu J. (2019). Assessing systemic and non-systemic transmission risk of tick-borne encephalitis virus in Hungary. PLoS ONE.

[81] Nah K., Bede-Fazekas Á., Trájer A.J., Wu J. (2020). The potential impact of climate change on the transmission risk of tick-borne encephalitis in Hungary. BMC Infect. Dis..

[82] Zeman P. (2019). Prolongation of tick-borne encephalitis cycles in warmer climatic conditions. Int. J. Environ. Res. Public Health.

[83] Randolph S.E., Rogers D.J. (2000). Fragile transmission cycles of tick-borne encephalitis virus may be disrupted by predicted climate change. Proc. R. Soc. B Biol. Sci..

[84] Sumilo D., Asokliene L., Bormane A., Vasilenko V., Golovljova I., Randolph S.E. (2007). Climate Change Cannot Explain the Upsurge of Tick-Borne Encephalitis in the Baltics. PLoS ONE.

[85] Lindgren E., Gustafson R. (2001). Tick-borne encephalitis in Sweden and climate change. Lancet.

[86] Daniel M., Danielová V., Fialová A., Malý M., Kříž B., Nuttall P.A. (2018). Increased relative risk of tick-borne encephalitis in warmer weather. Front. Cell. Infect. Microbiol..

[87] Kříž B., Kott I., Daniel M., Vráblík T. (2015). Beneš Vliv klimatických změn na výskyt onemocnění klíšťovou encefalitidou v letech 1982-2011 v České republice. Epidemiol. Mikrobiol. Imunol..

[88] Brabec M., Daniel M., Malý M., Danielová V., Kříž B., Kott I., Beneš Č. (2017). Analysis of meteorological effects on the incidence of tick-borne encephalitis in the Czech Republic over a thirty-year period. Virol. Res. Rev..

[89] Home—NPHO EOΔΥ. https://eody.gov.gr/en/.

[90] Tick-Borne Encephalitis. https://eody.gov.gr/disease/krotonogenis-egkefalitida/.

[91] Baylis M. (2017). Potential impact of climate change on emerging vector-borne and other infections in the UK. Environ. Health Glob. Access Sci. Source.

[92] Lukan M., Bullova E., Petko B. (2010). Climate warming and tick-borne encephalitis, Slovakia. Emerg. Infect. Dis..

[93] Mills J.N., Gage K.L., Khan A.S. (2010). Potential influence of climate change on vector-borne and zoonotic diseases: A review and proposed research plan. Environ. Health Perspect..

[94] Lindgren E., Andersson Y., Suk J.E., Sudre B., Semenza J.C. (2012). Public health: Monitoring EU emerging infectious disease risk due to climate change. Science.

[95] Ashraf U., Peterson A.T., Chaudhry M.N., Ashraf I., Saqib Z., Ahmad S.R., Ali H., Ashraf C., Peterson A.T., Chaudhry M.N. (2017). Ecological niche model comparison under different climate scenarios: A case study of Olea spp. in Asia. Ecosphere.

[96] Li S., Gilbert L., Harrison P.A., Rounsevell M.D.A. (2016). Modelling the seasonality of lyme disease risk and the potential impacts of a warming climate within the heterogeneous landscapes of Scotland. J. R. Soc. Interface.

[97] Boeckmann M., Joyner T.A. (2014). Old health risks in new places? An ecological niche model for I. ricinus tick distribution in Europe under a changing climate. Health Place.

[98] Raghavan R.K., Townsend Peterson A., Cobos M.E., Ganta R., Foley D. (2019). Current and Future Distribution of the Lone Star Tick, Amblyomma americanum (L.) (Acari: Ixodidae) in North America. PLoS ONE.

[99] Alkishe A.A., Peterson A.T., Samy A.M. (2017). Climate change influences on the potential geographic distribution of the disease vector tick Ixodes ricinus. PLoS ONE.

[100] Bregnard C., Rais O., Voordouw M.J. (2020). Climate and tree seed production predict the abundance of the European LB vector over a 15-year period. Parasites Vectors.

[101] Nolzen Id H., Brugger K., Reichold A., Brock J., Lange M., Thulkeid H.-H. (2022). Model-based extrapolation of ecological systems under future climate scenarios: The example of Ixodes ricinus ticks. PLoS ONE.

[102] Donša D., Grujić V.J., Pipenbaher N., Ivajnšič D. (2021). The lyme borreliosis spatial footprint in the 21st century: A key study of slovenia. Int. J. Environ. Res. Public Health.

[103] Uusitalo R., Siljander M., Dub T., Sane J., Sormunen J.J., Pellikka P., Vapalahti O. (2020). Modelling habitat suitability for occurrence of human tick-borne encephalitis (TBE) cases in Finland. Ticks Tick-Borne Dis..

